# Countrywide Reassessment of *Schistosoma mansoni* Infection in Burundi Using a Urine-Circulating Cathodic Antigen Rapid Test: Informing the National Control Program

**DOI:** 10.4269/ajtmh.16-0671

**Published:** 2017-03-08

**Authors:** Giuseppina Ortu, Onésime Ndayishimiye, Michelle Clements, Donatien Kayugi, Carl H. Campbell, Mariama Sani Lamine, Antonio Zivieri, Ricardo Soares Magalhaes, Sue Binder, Charles H. King, Alan Fenwick, Daniel G. Colley, Peter Mark Jourdan

**Affiliations:** 1 Schistosomiasis Control Initiative (SCI), Department of Infectious Disease Epidemiology, Imperial College London, United Kingdom.; 2 Programme National Intégré de lutte contre les Maladies Tropicales Négligées et la Cécité (PNIMTNC), Ministère de la Santé Publique et de la Lutte contre le SIDA, Bujumbura, Burundi.; 3 Schistosomiasis Consortium for Operational Research and Evaluation (SCORE), Department of Microbiology, Center for Tropical and Emerging Global Diseases (CTEGD), University of Georgia, Athens, Georgia.; 4 World Health Organization (WHO), Libreville, Gabon.; 5 School of Veterinary Science, The University of Queensland, Gatton Campus, Gatton, Australia.; 6 Children's Health Research Centre, The University of Queensland, South Brisbane, Australia.; 7 Center for Global Health and Diseases, Case Western Reserve University School of Medicine, Cleveland, Ohio.

## Abstract

Following implementation of the national control program, a reassessment of *Schistosoma mansoni* prevalence was conducted in Burundi to determine the feasibility of moving toward elimination. A countrywide cluster-randomized cross-sectional study was performed in May 2014. At least 25 schools were sampled from each of five eco-epidemiological risk zones for schistosomiasis. Fifty randomly selected children 13–14 years of age per school were included for a single urine-circulating cathodic antigen (CCA) rapid test and, in a subset of schools, for duplicate Kato-Katz slide preparation from a single stool sample. A total of 17,331 children from 347 schools were tested using CCA. The overall prevalence of *S. mansoni* infection, when CCA trace results were considered negative, was 13.5% (zone range [zr] = 4.6–17.8%), and when CCA trace results were considered positive, it was 42.8% (zr = 34.3–49.9%). In 170 schools, prevalence of this infection determined using Kato-Katz method was 1.5% (zr ==0–2.7%). The overall mean intensity of *S. mansoni* infection determined using Kato-Katz was 0.85 eggs per gram (standard deviation = 10.86). A majority of schools (84%) were classified as non-endemic (prevalence = 0) using Kato-Katz; however, a similar proportion of schools were classified as endemic when CCA trace results were considered negative (85%) and nearly all (98%) were endemic when CCA trace results were considered positive. The findings of this nationwide reassessment using a CCA rapid test indicate that *Schistosoma* infection is still widespread in Burundi, although its average intensity is probably low. Further evidence is now needed to determine the association between CCA rapid test positivity and low-intensity disease transmission.

## Introduction

More than 200 million people are infected with *Schistosoma* parasites and many more are thought to be at risk of infection.^1^ Increased advocacy, resources, and donor commitment, including substantial drug donations by pharmaceutical companies, have enabled the implementation of the World Health Organization (WHO) guidelines for the control of schistosomiasis in endemic countries.^1^ To move from control of morbidity toward elimination of infection, new diagnostic tools and evidence-based interpretation of test results are now needed to consistently inform national control programs.^2^

For several decades, the detection of *Schistosoma mansoni* eggs in slides prepared using the Kato-Katz technique has been the mainstay of diagnosis in field settings.^3^ Recently, the development and commercial availability of a field-friendly lateral flow rapid test for detection of circulating cathodic antigen (CCA) in urine has led to more convenient collection of parasitological data that, if validated, could provide useful information to schistosomiasis control managers.^4^ Studies suggest that the CCA rapid test is more sensitive than a single Kato-Katz test in the diagnosis of *S. mansoni* infection, especially in low-prevalence areas where most infections are of low intensity.^5^ However, further evidence is needed to understand how accurately CCA test results can detect ongoing low-level parasite transmission and how positive results should be interpreted as part of national control strategies.

In 2007, mapping of neglected tropical diseases (NTDs) in Burundi found that several of its regions were at risk of schistosomiasis and that *S. mansoni* was the only human schistosome species that was endemic.^6^ On the basis of the risk maps produced at the time, a national NTD control program was rolled out in which annual mass drug administration (MDA) of praziquantel targeted school-age children as well as pregnant mothers (in certain areas).^7^

In 2014, after 6 years of praziquantel annual mass distribution in targeted areas, and sentinel site monitoring data that indicated a decline in prevalence of infection (Supplemental Table 1), the national burden of schistosomiasis was reassessed to determine the feasibility of moving toward elimination of *S. mansoni* in Burundi. The geographical distribution of *S. mansoni* was determined using a urine-based CCA rapid cassette assay to test a selection of school-age children, complemented using Kato-Katz stool assays performed in a subset of schools. This article presents the results of this 2014 survey and discusses how these data can be interpreted to inform the national schistosomiasis control strategy.

## Materials and Methods

### Study site.

Burundi is a small, densely populated country in eastern Africa, with more than 420 people per kilometer.^8^ Close to 90% of the population live in rural areas, which may be divided into three main ecological zones: lakes, hills, and plateaus. On average, 75% of people have access to clean drinking water, but less than half of the population has access to sanitation facilities and there are large differences in access between rural and urban settings, with the lowest levels of access in rural areas.^8^ The majority of residents make a living through agriculture and nearly half of the population in Burundi is younger than 15 years of age.^8^

Kato-Katz testing in 2007 found that 24 of the country's 129 communes were at moderate risk of *S. mansoni* infection (i.e., defined by the WHO as school-age prevalence above 10%), and a further 63 communes were at low risk of the infection.^7^ On the basis of the annual mass praziquantel treatment reports provided by the Ministry of Health in Burundi, roughly 60–70% of school-age children were treated routinely once a year in areas with *S. mansoni* prevalence, which was estimated to be above 10% in 2007.

### Study design.

#### Determining mapping zones.

**Figure 1.**
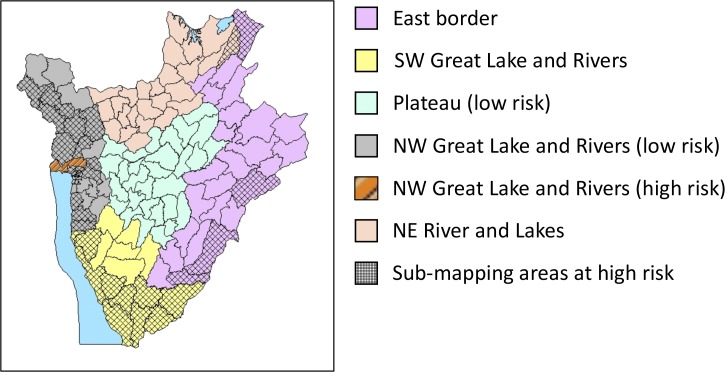
Eco-epidemiological risk zones of treatment implementation units (communes) for reassessment of *Schistosoma mansoni* control in Burundi. The map shows communes grouped within the five ecological zones (by color), with each ecological zone divided into two subareas: high-risk areas delineated by grid or striped patterns, and low-risk areas with no pattern. High-risk areas with *striped* pattern are those that during 2007 mapping were found to have communes with a predicted mean prevalence above 10% and an upper confidence interval above 50%; those with *grid* patterns are areas that in 2007 were found to have communes having a predicted mean prevalence below 10%, with an upper confidence interval between 10% and 50%. Low-risk areas: areas without pattern were found to have communes with a predicted mean prevalence below 10%, and an upper confidence interval below 10% in 2007.

A multistage, cluster-randomized, cross-sectional study was performed in May 2014. For prevalence reassessment, the country was divided into five eco-epidemiological zones based on 1) previous schistosomiasis risk maps and impact surveys performed during 2007–2011 in 31 sentinel site primary schools, and 2) their ecological settings, that is, lake, river, island, plain/plateau, hills and mountainous regions, peri-urban, and rural (see Figure 1

and Table 1).

#### Distribution of the CCA sampling effort.

Data collected in 2009 were used to estimate likely *S. mansoni* prevalence in the various ecological zones and to determine how best to allocate the sampling efforts to obtain the most meaningful results, given study resources available to test approximately 350 schools. For example, given the survey's resource limitations, a 1% effort was put into sampling schools in the northeast river and lakes and in the plateau zones, as the likelihood of identifying infected children was particularly low in those areas, according to the 2009 prevalence data. Our initial model suggested that only one school should be tested in the northeast river and lakes and in the plateau zones. However, because a primary purpose of this mapping was also to assess the feasibility of elimination of *S. mansoni* infection, the sample size in these low-risk zones was increased to 50 to ensure adequate prevalence data in the areas where elimination might be potentially most feasible. Table 1 shows the distribution of final sampling effort by zone as developed using this approach.

The five zones were divided into groups of “subzones” to be able to use survey results to define “implementation units” for further drug distribution. Each subzone was initially assigned a level of risk based on previously obtained epidemiological risk maps.^6^ “High risk” was assigned to communes with an upper 95% confidence interval (95% CI) of *S. mansoni* prevalence greater than 10% as determined using Kato-Katz testing, and “low risk” to those with an upper 95% CI of *S. mansoni* prevalence lower than 10%.

The number of primary schools sampled in each subzone was proportional to the total number of communes in each subzone, with communes in high-risk zones allocated proportionally twice as many schools to survey as communes in low-risk zones. This double stratification ensured a larger sample size in zones expected to have high transmission, but, conversely, a smaller sample size in zones expected to have low transmission.

The schools to be mapped using the urine CCA assay were randomly sampled from a list of all primary schools in the subzone, with no reference to school size. However, prior to random sampling, *purposive* sampling was performed in subzone areas where prevalence of *S. mansoni* infection was expected to be > 10% based on prior years' data (communes of Rugombo, Rumonge, Mpanda, and Nyanza Lac); further *random* sampling was then performed to adjust the number of schools to the number assigned to each zone. During data collection, three schools selected for mapping with CCA in northwestern Great Lakes and Rivers (two from northwestern high and one from northwestern low subzones) were not able to be visited, giving a total of 347 schools tested using the CCA method.

#### Kato-Katz sampling efforts.

Five schools per district in 45 districts were sampled for mapping both soil-transmitted helminthiasis and *S. mansoni* using the Kato-Katz technique. Where possible, Kato-Katz was performed in schools already selected for CCA testing. In districts with more than five schools selected for CCA testing, five schools were randomly selected from those for Kato-Katz testing. In districts with less than five schools to be mapped with CCA, schools to be mapped using Kato-Katz only were randomly selected from a list of all schools in the district. In addition, both CCA and Kato-Katz data were collected at 26 *sentinel site* schools (where monitoring of the national schistosomiasis control program had previously taken place) if the schools had not been randomly selected during the survey sampling. Supplemental Table 1 provides the Kato-Katz prevalence data for 2007–2011 for all 31 sentinel schools and the 26 tested in 2014, as well as the CCA prevalence data obtained at all 31 sentinel schools in 2014.

In total, 177 schools assigned to mapping using CCA testing were also selected for mapping with Kato-Katz testing. Kato-Katz data collected from 13 schools were discarded. During quality control, it was determined that data collection was not done according to protocol. Specifically, stool specimens were not read during the day and under good lighting conditions, hindering the ability to obtain reliable egg counts. Eight of these schools were from the northeastern low subzone and five were from the plateau zone and subzone. However, there remained six schools selected for testing with CCA only that also had Kato-Katz data collected (five from northwestern high subzone and one from southwestern low subzone). We included Kato-Katz data from these six schools in the results, giving a total of 170 schools assessed using both CCA and Kato-Katz testing.

#### Sampling of children within schools.

On the basis of the ages of children attending primary schools in Burundi, in each school, 50 randomly selected children of 13 and 14 years of age attending school the day of the survey were included for parasitological testing. In schools with fewer than 50 children aged 13–14 years, randomly selected 15-year-olds were added to the survey group until 50 children were included. If necessary, children aged 12 and 16 years were also randomly added until 50 children were included for parasitological data collection.

### Parasitological data collection.

A single urine sample collected from each included child was tested with a single urine CCA rapid test according to the manufacturer's instructions (Rapid Medical Diagnostics, Pretoria, South Africa). Using a single-use pipette, one drop of urine was added to the cassette test well, followed by a drop of the test reagent. Technicians read the test results after exactly 20 minutes under good lighting conditions. For grading of the CCA test intensities as 0 = negative; 1 = trace; 2 = +; 3 = ++; 4 = +++, each laboratory team was given a reference image of four CCA tests with the incremental band readings shown.^9^ Any uncertainty in interpretation of the test results was discussed and resolved immediately with a trained senior laboratory technician.

In sites selected for stool examination, a single stool sample was collected from each of the included children and duplicate slides were prepared using the Kato-Katz technique.^3^ A standard kit was used to prepare slides for microscopic examination and the number of *S. mansoni* eggs per slide were counted and recorded.

For the first 10 sites visited by the eight respective survey teams, 20 of the Kato-Katz slides were reread for quality control by an experienced microscopist. If more than 10 of the slide results were discordant between the lead microscopist and either one or both of the laboratory technicians, additional training was provided on site to improve the quality of microscopy. If slide results were discordant at the first 10 surveyed schools, 10% of the Kato-Katz slides in the remaining survey sites were reread by the lead microscopist for quality control. Any discordant results were discussed and resolved on site.

### Data entry and statistical analysis.

Stool data were collected on paper forms and CCA results were collected on paper forms and data capture forms on Nexus 7 tablets with software developed through Open Data Kit (ODK) LINKS created by the Task Force for Global Health (http://www.taskforce.org). Summary data for the CCA results from the tablets were communicated on the same day as data collection, or as soon as an internet connection could be established, to the central level for review of results and follow-up action if needed. Once the field work was completed, the data were double entered into an Excel spreadsheet (Microsoft Corporation, Redmond, WA), and all discrepancies were resolved.

Statistical analysis was performed using R version 3.1.2 (The R Foundation, Vienna, Austria). Prevalence of infection was defined as the number of children infected divided by the number of children tested. CCA results when trace readings were considered as negative are reported as “CCA trace negative,” and results when trace readings were considered as positive are reported as “CCA trace positive.”

For schools mapped using Kato-Katz testing, prevalence was defined as the number of children with *S. mansoni* eggs on any slide prepared from their stool divided by the number of children tested. *Schistosoma mansoni* eggs per gram (epg) of stool were calculated for each slide by multiplying the number of eggs per slide by 24. The mean intensity of infection for each individual was determined by calculating the arithmetic mean epg of all slides sampled per individual. Light-intensity *S. mansoni* infection was defined as 1–99 epg, moderate-intensity infection as 100–399 epg, and heavy-intensity infection as > 399 epg.^10^

The study was designed to estimate prevalence of *S. mansoni* infection in each zone separately, and not at the country level. Thus, we report the results overall and also split by zone for ease for interpretation. As the survey was clustered, we report measures of variability based on the cluster level below that being reported, rather than at the individual level. For overall measurements, we report the full range of zone measurements (as there were only five zones and outliers at this level were of specific interest to program planners). For zone-level measurements, we report the interquartile range of the village estimates, at levels where outliers are of less programmatic interest.

Prevalence of *S. mansoni* infection was analyzed using generalized linear mixed models implemented in lme4 in R software.^11^ Age, standardized by subtracting the mean and dividing by the standard deviation, and its associated quadratic, sex, and either zone or subzone were included as fixed effects, and school was included as the only random effect. We used the zone plateau as the reference zone in the multivariable model, as all areas within this zone were believed to have low prevalence.^7^

### Ethical considerations.

This survey was granted approval by the Ministry of Health in Burundi and the Institutional Review Board of Imperial College London (St Mary Research Ethics Committee of Imperial College, UK, 2003 /EC No 03.36, R&D No: 03/SB/003E, amended in 2007 /REC Ref: AM01, May 2007). The survey data were collected as an intrinsic part of the activities of the Ministry of Public Health in Burundi and had minimal risk for harm to any of the participants. Head teachers and parents of the schoolchildren were informed about the objectives and expected benefits of the survey, and free, informed written consent was provided by the head teacher on behalf of the schoolchildren for the collection of single urine and stool samples from each selected child. Prior to sample collection, the head teacher informed all eligible children of the objectives and expected benefits of the survey, and each child was free to withdraw at any time of the survey without consequences.

Individual child names were registered on paper forms only to ensure unique sample collection and to ensure individual anti-helminthic treatment if necessary. Children found to be test positive received treatment either on the spot or during an MDA occurring within a couple of weeks (in areas eligible for MDA). The participants' names were not entered into the survey database, and the paper forms were securely archived by the Ministry of Health.

## Results

A total of 17,331 children from 347 schools selected for this survey provided single urine samples for the detection of *S. mansoni* infection using CCA method.

### CCA mapping results.

**Figure 2.**
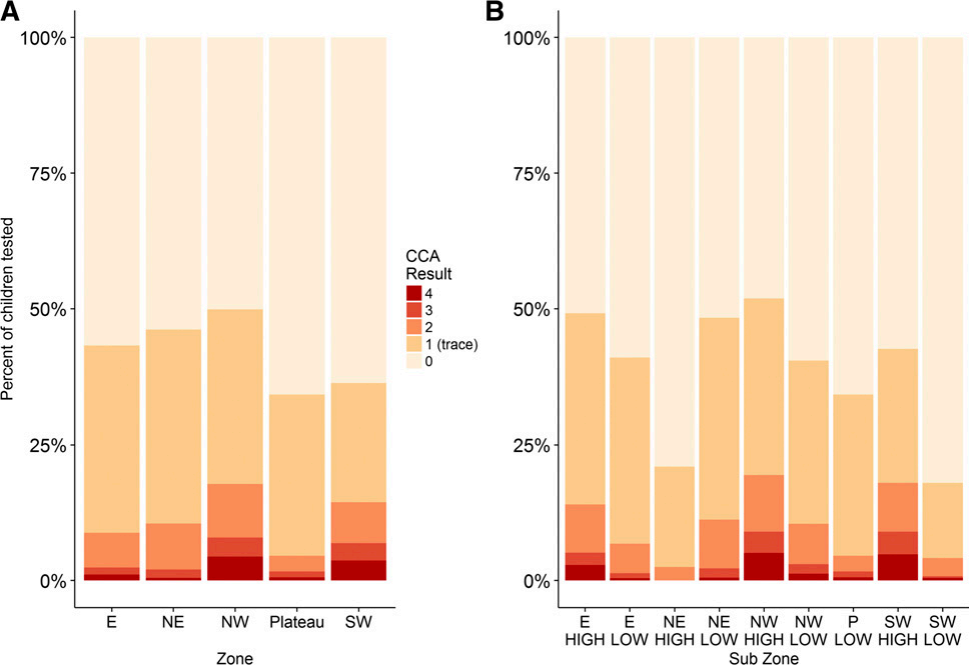
Circulating cathodic antigen (CCA) test results by zone (**A**) and subzone (**B**) in 347 schools mapped by CCA. CCA 0 indicates a negative test, CCA 1 indicates a trace result, CCA 2 indicates a 1+ reading, CCA3 indicates a 2+ reading, and CCA4 indicates a 3+ reading.

As shown in Table 2 and Figure 2

the overall prevalence of *S. mansoni* infection using CCA with trace considered as negative was 13.5% (zone range [zr] = 4.6–17.8%), rising to 42.8% (zr = 34.3–49.9%), if CCA trace was considered to be positive. Eighty-five per cent of schools were CCA trace negative (zr = 72.5–91.0%), whereas 98.6% of schools were CCA trace positive (zr = 92.5–100.0%).

As shown in Tables 3 and 4, prevalence of *S. mansoni* infection was significantly higher among boys, with the difference being most pronounced when CCA trace was considered negative. Also, a significant negative association was observed with older age of being test positive by either CCA trace negative or CCA trace positive criteria.

### Comparison of prevalence of *S. mansoni* infection using CCA.

Figure 3

**Figure 3.**
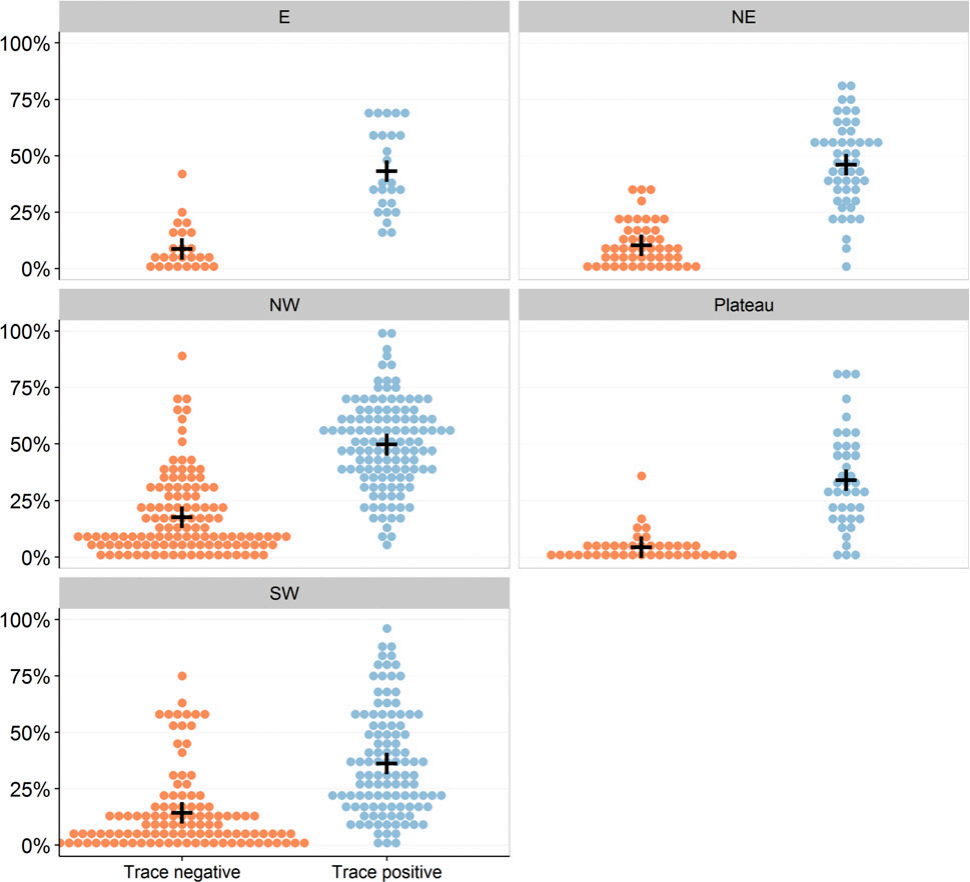
Circulating cathodic antigen (CCA) test results by zone in all 347 CCA-mapped schools. Each dot represents an individual school prevalence of *Schistosoma mansoni* infection, and black crosses represent the mean zone prevalence by CCA. The individual panels present results for the different study zones. Left-sided orange dot clusters represent prevalence when CCA trace values are considered to be negative. Right-sided blue dot clusters represent prevalence when CCA trace values are considered to be positive.

shows prevalence of *S. mansoni* infection using CCA trace negative and CCA trace positive criteria. As expected, prevalence of *S. mansoni* infection in the plateau zone was the lowest of all the zones at 4.6% by CCA trace negative and 34.4% by CCA trace positive criteria (Table 2). As shown in Table 3, prevalence of *S. mansoni* infection using CCA trace negative criteria was significantly higher in all zones (prevalence ≥ 10.5%) compared with the plateau zone, except for the eastern zone (prevalence = 8.8%) where the difference was marginally nonsignificant (*P* = 0.05). However, when CCA trace was considered as positive, only the northeastern and northwestern zones (prevalence ≥ 46.2%) had significantly higher prevalence than the plateau zone.

Analysis of subzone prevalence showed that prevalence of *S. mansoni* infection in the southwestern high and northwestern high zones was significantly higher than that in the plateau zone, using both CCA trace negative (> 19.0%) and CCA trace positive (> 42.7%) criteria (Figure 2, Table 4). The prevalence of *S. mansoni* infection in the north eastern zone was significantly higher than that in the plateau using CCA trace negative results, but this difference was not significant in comparing CCA trace positive results.

The marginal *R*^2^ (considering fixed effects only) was low for all the multivariable models (< 11%) indicating further unexplained variation in the observed prevalence data. However, the conditional *r*^2^ (considering the random effect of schools) was higher (> 26%), reflecting that some of the variation could be explained by differences in prevalence among schools, that is, at the community level.

### Comparison of prevalence of *S. mansoni* infection using CCA and Kato-Katz.

A total of 8,482 children from 170 schools provided both a single stool and a single urine sample for analysis using both Kato-Katz in stool and CCA rapid test in urine (Table 5 ). Prevalence of *S. mansoni* infection by CCA in the subset of children analyzed using Kato-Katz was similar to, but slightly lower than, prevalence in all mapping schools. The overall prevalence by CCA trace negative was 10.9% (zr = 4.0–13.5%), rising to 41.3% (zr = 34.4–46.7%) by CCA trace positive criteria.

Prevalence of *S. mansoni* infection determined using Kato-Katz in the 170 schools was much lower than that determined using CCA at 1.5% (zr = 0–2.7%). The overall mean intensity of *S. mansoni* infection by Kato-Katz was 0.85 epg, and only one child had heavy-intensity infection. No infections were identified using Kato-Katz in the plateau zone, whereas the prevalence determined using CCA in the same children was 4.6% by CCA trace negative and 34.3% by CCA trace positive criteria (Table 5).

**Figure 4.**
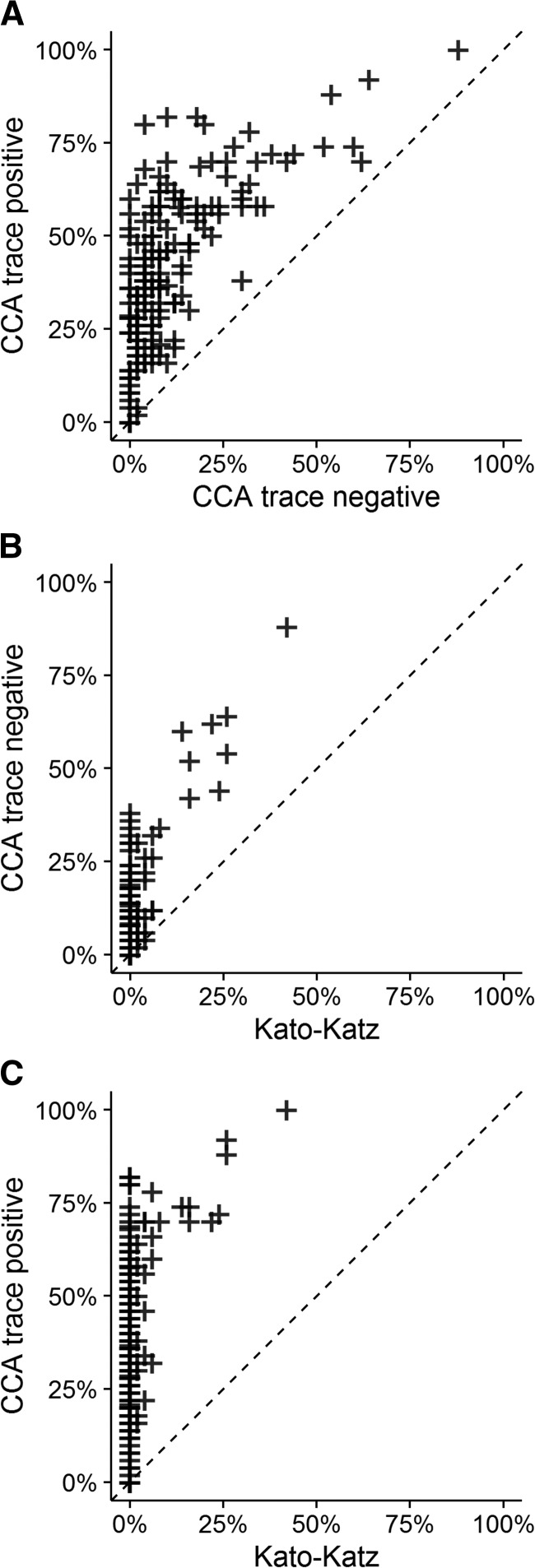
Scatterplot comparisons of school prevalence of *Schistosoma mansoni* infection as measured by different test criteria in 170 schools. Prevalence of *S. mansoni* infection as measured by (**A**) circulating cathodic antigen (CCA) trace positive vs. CCA trace negative; (**B**) CCA trace negative vs. Kato-Katz; and (**C**) CCA trace positive vs. Kato-Katz.

A majority of schools (84%) did not have schistosome infections detected (prevalence = 0) by Kato-Katz. The prevalence determined using CCA in these “Kato-Katz-negative” schools ranged from 0.0% to 38.0% with a mean of 7.7% by CCA trace negative, and 0.0–82.0% with a mean of 38.2% by CCA trace positive criteria (Figure 4

). The prevalence of *S. mansoni* infection determined using Kato-Katz was never greater than that seen using CCA. Among the 27 schools where *S. mansoni* eggs were detected by Kato-Katz, prevalence ranged from 2.0% to 88.0%, with a mean of 27.8%, by CCA trace negative criteria, and 16.0% to 100.0%, with a mean of 57.4%, by CCA trace positive criteria. Of the 10% schools with the highest prevalence by CCA trace negative, 10 of 18 schools (55.6%) were also in the 10% schools with the highest prevalence by Kato-Katz. For the 10% of schools with the highest prevalence by CCA trace positive, 12 of 19 (63.2%) were also in the 10% of schools with the highest prevalence by Kato-Katz.

## Discussion

In an increasing number of endemic countries, national schistosomiasis control programs have provided mass distribution of praziquantel to at-risk populations for multiple years.^1^,^7^ In some countries, such as in Burundi, control strategies have successfully reduced the prevalence of clinical schistosomiasis. Because the WHO and partners are now currently aiming to eliminate *Schistosoma* infection in selected countries in Africa,^12^ improved diagnostic tools, in terms of both performance and convenience for field surveys, are needed for countries that propose to move toward elimination of *Schistosoma* infections.^2^

The development of the urine CCA rapid test has provided promising means to determine the distribution of *S. mansoni* infection, especially in areas where the disease burden is largely below the detection level of the conventional Kato-Katz technique.^5^,^13^ However, further evidence is needed to determine how CCA rapid test results relate to detection of continuing parasite transmission and the persistence of subtle morbidities caused by low-intensity infections. Such data will guide the interpretation of CCA rapid test results in those formerly highly endemic countries that now want to consider elimination.

In line with previous findings, this survey found a significantly higher prevalence of *S. mansoni* infection using urine CCA rapid test as opposed to conventional microscopy of duplicate Kato-Katz slides.^5^ Microscopic detection of *Schistosoma* eggs by Kato-Katz technique is prone to fluctuations in egg excretion as well as uneven distribution of eggs within each stool sample.^14^ The sensitivity of Kato-Katz technique has been shown to be especially low in areas with low prevalence of *S. mansoni* infection. Although CCA rapid test results in both serum and urine have been found to fluctuate, the diurnal variation is less pronounced than that of egg excretion as detected by the Kato-Katz technique.^15^–^17^

The large number of CCA trace values detected in this survey require an appropriate determination of their significance in interpreting CCA rapid test results, if CCA results are to provide useful data to inform control strategies. Previous studies have found a high overall sensitivity for *S. mansoni* infection using the CCA rapid test.^4^,^5^,^9^,^14^,^17^ Particularly in low-prevalence areas, many of the CCA positive results are likely to be trace positive. As would be expected when compared with a highly specific, low-sensitivity assay, the diagnostic test specificity of CCA, as compared with standard microscopy, seems low.^17^,^18^ However, these trace results most probably represent true infections that are not detected by the Kato-Katz technique,^14^,^17^ and it is the high false-negative rate of Kato-Katz testing that wrongly skews the simple classification of CCA specificity. Some CCA trace positives may be false-positives through misreading of test results; most likely they are a mix of these two possibilities. From a resource perspective, we would argue that further evidence is needed to validate the CCA rapid test results' relationship with risk of parasite transmission, especially in low-prevalence areas, so that recommendations can be provided on the use of trace results in defining new national treatment targets.

These findings for Burundi suggest the need for additional control measures, beyond preventive chemotherapy through praziquantel MDA, to achieve maximum reductions of *Schistosoma* infection in Burundi.^19^ Studies have shown that improved access to safe water, hand-washing facilities, and latrines are associated with reduced transmission of *S. mansoni* infection, and it is predicted that true elimination of disease will not be feasible without concurrent improvement in living conditions for populations at risk.^20^,^21^

This reassessment of *S. mansoni* prevalence was done to inform potential development of an elimination strategy in Burundi. If the Ministry of Health were to follow the Kato-Katz results and current WHO guidelines for morbidity control,^11^ no further MDA would be indicated. Going against this, the CCA results, even with traces analyzed as negative, suggest that *Schistosoma* infection is still widespread, although more evidence is needed to determine how these prevalence results reflect the individual and collective worm burden and egg output in affected communities.

Potential limitations of this comparative mapping survey include, as with all such studies on schistosomiasis, the lack of a gold standard for the diagnosis. Consequently, it is not possible to determine a true prevalence in the population. Furthermore, only a single stool per student was assayed using the Kato-Katz technique, and the prevalence results by stool microscopy may have been prone to the interobserver variability as well as low sensitivity of the test, in this low-prevalence and low-intensity population. In this survey, a photograph of each of the possible test results was provided to each of the survey teams to assist in interpretations of CCA test results and the test results were read by a single person on each survey team. However, there may have been differences among readings by different assessors due to technical skills, lighting, and visual interpretations of the test bands. Another potential limitation is that only children attending school at the day of the testing were included in this survey, potentially introducing a selection bias.

The geographically focal distribution as well as the low prevalence of *S. mansoni* infection in Burundi made the sampling design challenging. We aimed to include a sample size that would be representative of as small an area as possible, yet in line with the implementation units for the national schistosomiasis control program. The results of this study are therefore representative for groups of communes, rather than a more ideal high-resolution mapping of smaller units of these regions.

The analysis of CCA rapid test results presented in this article should be repeated in other countries endemic for *S. mansoni* to provide further evidence for defining antigen-prevalence thresholds for selecting control interventions. It is possible that revision of control guidelines might need to be done on a country-to-country basis, in regard to country-based programmatic goals, once CCA rapid test performance is assessed with regard to local risk of parasite transmission and risk of persisting morbidity. In the meantime, the results in this article may provide guidance to prioritize the allocation of praziquantel MDAs to specific regions of Burundi.

The findings of this nationwide reassessment of *S. mansoni* infection using CCA rapid test indicate that, in Burundi, infection is still widespread, although the average intensity of infection is probably low. This study contributes to the body of CCA and Kato-Katz comparative data in low-intensity settings, and its results suggest that the schistosomiasis prevention strategy needs to be revised taking into consideration the reduced prevalence of *Schistosoma* infections.

## Supplementary Material

Supplemental table.

## Figures and Tables

**Table 1:** Numbers of primary schools mapped with CCA per zone and subzone

Zone	Subzone	Number of schools per zone	Number of schools per subzone
East border (E)	E-high	25	7
	E-low		18
Northeast Rivers and Lakes (NE)	NE-high	50	4
	NE-low		46
Northwest Great Lakes and Rivers	NW-high	122*	100
	NW-low		22
Plateau	High	40	0
	Low		40
Southwest Great Lakes and Rivers (SW)	SW-high	110	82
	SW-low		28
**Total**		**347**	

* The initial protocol called for 125 schools to be sampled in the northwest Great Lakes and Rivers zone.

**Table 2:** Characteristics of 347 schools mapped with CCA*

	Zone					
	All zones	East	Northeast	Northwest	Plateau	Southwest
Number of schools	347	25	50	122	40	110
Number of children	17,331	1,248	2,500	6,092	1,992	5,499
Percentage of girls (excluding missing)	50.0	49.9	50.0	50.0	50.3	50.0
Number of missing sex data	152	0	101	0	50	1
Mean age in years (SD)	13.19 (0.86)	13.28 (0.68)	13.37 (0.72)	13.08 (0.91)	13.40 (0.68)	13.12 (0.92)
Number missing age	8	0	1	3	0	4
Number CCA 0*	9,913	707	1,346	3,054	1,309	3,497
Number CCA 1 (trace)	5,074	431	891	1,953	592	1,207
Number CCA 2	1,366	80	212	601	58	415
Number CCA 3	467	16	39	215	21	176
Number CCA 4	511	14	12	269	12	204
Percentage of children with infection: CCA trace negative (school IQR)	13.5 (2.0–18.0)	8.8 (2.0–14.0)	10.5 (2.5–16.0)	17.8 (6.0–28.0)	4.6 (0.0–6.0)	14.5 (2.5–16.0)
Percentage of children with infection: CCA trace positive (school IQR)	42.8 (26.0–58.0)	43.3 (28.0–60.0)	46.2 (16.0–34.5)	49.9 (38.0–62.0)	34.3 (16.0–48.0)	36.4 (18.0–52.0)
Percentage of schools with any infections: CCA trace considered negative	85.3	88.0	82.0	91.0	72.5	84.5
Percentage of schools with any infections: CCA trace considered positive	98.6	100.0	100.0	100.0	92.5	98.2

CCA = circulating cathodic antigen point-of-care urine cassette assay; IQR = interquartile range; SD = standard deviation of the mean.

* CCA 0 indicates a negative test, CCA 1 indicates a trace result, CCA 2 indicates a 1+ reading, CCA 3 indicates a 2+ reading, CCA 4 indicates a 3+ reading.

**Table 3:** Logistic regression analysis of prevalence by CCA trace negative and CCA trace positive criteria, with zones analyzed as fixed effects

All CCA mapping schools		CCA trace negative			CCA trace positive		
Analysis by zone		17,172 pupils; 344 schools			17,172 pupils; 344 schools		
		*R*^2^_marginal_ = 0.059/*R*^2^_conditional_ = 0.386			*R*^2^_marginal_ = 0.026/*R*^2^_conditional_ = 0.254		
		Parameter	Adjusted odds ratio		Parameter	Adjusted odds ratio	
Fixed effects	Category	(SE)	(95% CI)	*P*	(SE)	(95% CI)	*P*
(Intercept)		−3.85 (0.26)	0.02 (0.01, 0.04)	< 0.001	−0.81 (0.17)	0.45 (0.32, 0.62)	< 0.001
Zone	Plateau						
	E	0.76 (0.39)	2.14 (1, 4.58)	0.050	0.46 (0.27)	1.58 (0.93, 2.68)	0.091
	NE	0.96 (0.33)	2.61 (1.37, 4.97)	0.004	0.55 (0.23)	1.74 (1.11, 2.72)	0.015
	NW	1.64 (0.28)	5.17 (2.97, 9.02)	< 0.001	0.78 (0.2)	2.17 (1.48, 3.19)	< 0.001
	SW	1.24 (0.29)	3.44 (1.96, 6.05)	< 0.001	0.09 (0.2)	1.09 (0.74, 1.61)	0.665
Age		−0.04 (0.03)	0.97 (0.91, 1.02)	0.246	−0.02 (0.021)	0.98 (0.94, 1.02)	0.314
Age^2^		−0.04 (0.02)	0.96 (0.92, 1)	0.027	−0.05 (0.014)	0.95 (0.93, 0.98)	< 0.001
Sex	F						
	M	0.48 (0.05)	1.62 (1.47, 1.79)	< 0.001	0.16 (0.034)	1.17 (1.1, 1.25)	< 0.001
Random effects		Variance	SD		Variance	SD	
School (intercept)		1.75	1.32		1.00	1.00	

CI = confidence interval; E = east; F = female; M = male; NE = northeast; NW = northwest; SD = standard deviation of the mean; SE = standard error of the mean; SW = southwest.

**Table 4:** Logistic regression analysis of prevalence using CCA trace negative and CCA trace positive, respectively, with subzones analyzed as fixed effects

All CCA mapping schools		CCA trace negative			CCA trace positive		
Analysis by subzone		17,172 pupils; 344 schools			17,172 pupils; 344 schools		
		*R*^2^_marginal_= 0.107/*R*^2^_conditional_ = 0.389			*R*^2^_marginal_ = 0.062/*R*^2^_conditional_ = 0.256		
		Parameter	Adjusted odds ratio		Parameter	Adjusted odds ratio	
Fixed effects	Category	(SE)	(95% CI)	*P*	(SE)	(95% CI)	*P*
(Intercept)		−3.81 (0.24)	0.02 (0.01, 0.04)	< 0.001	−0.8 (0.16)	0.45 (0.33, 0.61)	< 0.001
Zone	Plateau						
	E-high	1.33 (0.56)	3.79 (1.26, 11.39)	0.018	0.71 (0.4)	2.02 (0.92, 4.43)	0.078
	E-low	0.5 (0.41)	1.64 (0.74, 3.68)	0.226	0.35 (0.28)	1.42 (0.82, 2.45)	0.211
	NE-high	−0.43 (0.82)	0.65 (0.13, 3.24)	0.601	−0.79 (0.53)	0.45 (0.16, 1.28)	0.137
	NE-low	1.05 (0.32)	2.86 (1.54, 5.31)	0.001	0.66 (0.22)	1.94 (1.27, 2.96)	0.002
	NW-high	1.79 (0.27)	5.98 (3.49, 10.24)	< 0.001	0.86 (0.19)	2.37 (1.64, 3.41)	< 0.001
	NW-low	0.79 (0.38)	2.2 (1.04, 4.66)	0.039	0.33 (0.26)	1.39 (0.83, 2.32)	0.213
	SW-high	1.6 (0.28)	4.96 (2.86, 8.61)	< 0.001	0.42 (0.19)	1.53 (1.05, 2.22)	0.027
	SW-low	−0.15 (0.38)	0.86 (0.41, 1.79)	0.688	−0.99 (0.25)	0.37 (0.23, 0.61)	< 0.001
Age		−0.04 (0.03)	0.96 (0.91, 1.02)	0.213	−0.02 (0.021)	0.98 (0.94, 1.02)	0.267
Age^2^		−0.04 (0.02)	0.96 (0.92, 1)	0.03	−0.05 (0.014)	0.96 (0.93, 0.98)	0.001
Sex	F						
	M	0.48 (0.05)	1.62 (1.47, 1.79)	< 0.001	0.16 (0.034)	1.17 (1.1, 1.25)	< 0.001
Random effects		Variance	SD		Variance	SD	
School (intercept)		1.52	1.23		0.86	0.93	

CCA = circulating cathodic antigen point-of-care urine cassette assay; CI = confidence interval; E = east; F = female; M = male; NE = northeast; NW = northwest; SD = standard deviation; SE = standard error of the mean; SW = southwest.

**Table 5:** Characteristics of schools mapped by CCA* and Kato-Katz, overall and by zone

Zone	ALL	E	NE	NW	Plateau	SW
Number of schools	170	25	33	55	31	26
Number of pupils	8,482	1,248	1,650	2,742	1,542	1,300
Proportion girls (excluding missing)	50.0	49.9	50.2	49.7	50.3	50.1
Number missing sex	51	0	0	0	50	1
Mean age (SD)	13.25 (0.81)	13.28 (0.68)	13.42 (0.73)	13.13 (0.86)	13.42 (0.70)	13.07 (0.93)
Number missing age	0	0	0	0	0	0
Number CCA 0*	4,981	707	955	1,461	1,012	846
Number CCA 1 (trace)	2,574	431	532	863	469	279
Number CCA 2	588	80	132	249	40	87
Number CCA 3	175	16	23	77	18	41
Number CCA 4	164	14	8	92	3	47
Number KK uninfected	8,353	1,237	1,648	2,668	1,542	1,258
Number KK light infection	110	9	1	63	0	37
Number KK moderate infection	18	2	1	11	0	4
Number KK heavy infection	1	0	0	0	0	1
Percentage of children with infection: CCA trace negative (school IQR)	10.9 (2.0–14.0)	8.8 (2.0−14.0)	9.9 (2.0−16.0)	15.2 (4.0–21.0)	4.0 (0.0–6.0)	13.5 (2.0–13.5)
Percentage of children with infection: CCA trace positive (school IQR)	41.3 (26.0–58.0)	43.3 (28.0–60.0)	42.1 (30.0–56.0)	46.7 (36.0–59.0)	34.4 (16.0–49.0)	34.9 (18.0–47.5)
Percentage of children with infection: Kato-Katz (school IQR)	1.5 (0.0–0.0)	0.9 (0.0–0.0)	0.1 (0.0–0.0)	2.7 (0.0–1.0)	0.0 (0.0–0.0)	3.2 (0.0––4.0)
Mean intensity of infection: KK (epg; SD)	0.85 (10.86)	0.57 (8.07)	0.12 (3.94)	1.44 (12.29)	0 (0)	1.84 (19.13)
Percentage of schools with infection: CCA trace negative	82.4	88.0	81.8	89.1	71.0	76.9
Percentage of schools with infection: CCA trace positive	97.6	100.0	100.0	100.0	90.3	96.2
Percentage of schools with infection: Kato-Katz	15.9	12.0	3.0	25.5	0.0	34.6

CCA = circulating cathodic antigen point-of-care urine cassette assay; epg = eggs per gram; KK = Kato-Katz stool assay; SD = standard deviation of the mean; IQR = interquartile range.

* CCA 0 indicates a negative test, CCA 1 indicates a trace result, CCA 2 indicates a 1 + reading, CCA 3 indicates a 2 + reading, CCA 4 indicates a 3 + reading.

## References

[1] Fenwick A, Webster JP, Bosque-Oliva E, Blair L, Fleming FM, Zhang Y, Garba A, Stothard JR, Gabrielli AF, Clements AC, Kabatereine NB, Toure S, Dembele R, Nyandindi U, Mwansa J, Koukounari A (2009). The Schistosomiasis Control Initiative (SCI): rationale, development and implementation from 2002–2008. Parasitology.

[2] Hollingsworth TD, Adams ER, Anderson RM, Atkins K, Bartsch S, Basanez MG, Behrend M, Blok DJ, Chapman LA, Coffeng L, Courtenay O, Crump RE, de Vlas SJ, Dobson A, Dyson L, Farkas H, Galvani AP, Gambhir M, Gurarie D, Irvine MA, Jervis S, Keeling MJ, Kelly-Hope L, King C, Lee BY, Le Rutte EA, Lietman TM, Ndeffo-Mbah M, Medley GF, Michael E, Pandey A, Peterson JK, Pinsent A, Porco TC, Richardus JH, Reimer L, Rock KS, Singh BK, Stolk W, Swaminathan S, Torr SJ, Townsend J, Truscott J, Walker M, Zoueva A, Consortium NTDM (2015). Quantitative analyses and modelling to support achievement of the 2020 goals for nine neglected tropical diseases. Parasit Vectors.

[3] Katz N, Chaves A, Pellegrino J (1972). A simple device for quantitative stool thick-smear technique in schistosomiasis mansoni. Rev Inst Med Trop Sao Paulo.

[4] Lamberton PH, Kabatereine NB, Oguttu DW, Fenwick A, Webster JP (2014). Sensitivity and specificity of multiple Kato-Katz thick smears and a circulating cathodic antigen test for *Schistosoma mansoni* diagnosis pre- and post-repeated-praziquantel treatment. PLoS Negl Trop Dis.

[5] Colley DG, Binder S, Campbell C, King CH, Tchuem Tchuente LA, N'Goran EK, Erko B, Karanja DM, Kabatereine NB, van Lieshout L, Rathbun S (2013). A five-country evaluation of a point-of-care circulating cathodic antigen urine assay for the prevalence of *Schistosoma mansoni*. Am J Trop Med Hyg.

[6] Clements AC, Deville MA, Ndayishimiye O, Brooker S, Fenwick A (2010). Spatial co-distribution of neglected tropical diseases in the east African great lakes region: revisiting the justification for integrated control. Trop Med Int Health.

[7] Ndayishimiye O, Ortu G, Soares Magalhaes RJ, Clements A, Willems J, Whitton J, Lancaster W, Hopkins A, Fenwick A (2014). Control of neglected tropical diseases in Burundi: partnerships, achievements, challenges, and lessons learned after four years of programme implementation. PLoS Negl Trop Dis.

[8] World Bank (2015). Burundi.

[9] Stothard JR, Kabatereine NB, Tukahebwa EM, Kazibwe F, Rollinson D, Mathieson W, Webster JP, Fenwick A (2006). Use of circulating cathodic antigen (CCA) dipsticks for detection of intestinal and urinary schistosomiasis. Acta Trop.

[10] WHO (2011). Helminth Control in School-Age Children. A Guide for Managers of Control Programmes.

[11] Bates D, Machler M, Bolker B, Walker S (2015). Fitting linear mixed-effects models using lme4. J Stat Softw.

[12] WHO (2012). Accelerating Work to Overcome the Global Impact of Neglected Tropical Diseases. A Roadmap for Implementation.

[13] Kittur N, Castleman JD, Campbell CH, King CH, Colley DG (2016). Comparison of *Schistosoma mansoni* prevalence and intensity of infection, as determined by the Circulating Cathodic Antigen urine assay or by the Kato-Katz fecal assay: a systematic review. Am J Trop Med Hyg.

[14] Krauth SJ, Coulibaly JT, Knopp S, Traore M, N'Goran EK, Utzinger J (2012). An in-depth analysis of a piece of shit: Distribution of *Schistosoma mansoni* and hookworm eggs in human stool. PLoS NTD.

[15] Van Etten L, Engels D, Krijger FW, Nkulikyinka L, Gryseels B, Deelder AM (1996). Fluctuation of schistosome circulating antigen levels in urine of individuals with *Schistosoma mansoni* infection in Burundi. Am J Trop Med Hyg.

[16] Polman K, Engels D, Fathers L, Deelder AM, Gryseels B (1998). Day-to-day fluctuation of schistosome circulating antigen levels in serum and urine of humans infected with *Schistosoma mansoni* in Burundi. Am J Trop Med Hyg.

[17] Mwinzi PN, Kittur N, Ochola E, Cooper PJ, Campbell CH, King CH, Colley DG (2015). Additional evaluation of the Point-of-Contact Circulating Cathodic Antigen assay for *Schistosoma mansoni* infection. Front Public Health.

[18] Ochodo EA, Gopalakrishna G, Spek B, Reitsma JB, van Lieshout L, Polman K, Lamberton P, Bossuyt PM, Leeflang MM (2015). Circulating antigen tests and urine reagent strips for diagnosis of active schistosomiasis in endemic areas. Cochrane Database Syst Rev.

[19] WHO (2015). Investing to Overcome the Global Impact of Neglected Tropical Diseases. Third WHO Report on Neglected Tropical Diseases.

[20] Grimes JE, Croll D, Harrison WE, Utzinger J, Freeman MC, Templeton MR (2015). The roles of water, sanitation and hygiene in reducing schistosomiasis: a review. Parasit Vectors.

[21] Campbell SJ, Savage GB, Gray DJ, Atkinson JA, Soares Magalhaes RJ, Nery SV, McCarthy JS, Velleman Y, Wicken JH, Traub RJ, Williams GM, Andrews RM, Clements AC (2014). Water, Sanitation, and Hygiene (WASH): a critical component for sustainable soil-transmitted helminth and schistosomiasis control. PLoS Negl Trop Dis.

