# Supplementary material for: Countrywide Reassessment of Schistosoma mansoni Infection in Burundi Using a Urine-Circulating Cathodic Antigen Rapid Test: Informing the National Control Program
Source: Am J Trop Med Hyg. 2017 Mar 8;96(3):664–73. doi: 10.4269/ajtmh.16-0671 (PMC5361543; doi:10.4269/ajtmh.16-0671)
Supplement: Supplementary file 1 [file SD9.pdf]

Supplemental Table 1

Prevalence of *Schistosoma mansoni* infection in Burundian primary schools

Province (*N* = 17, as per 2012 administrative units)	District (*N* = 23, as per 2012 administrative units)	Commune (*N* = 27)	Study	School (*N* = 31)	School-level prevalence by Kato-Katz (%)						School-level prevalence by CCA (%)	
					2007	2008	2009	2010	2011	2014	2014 trace negative	2014 trace positive
Bubanza	Bubanza	Musigati	Pilot	Ruzibira	1.8	0	13.5	0	0.3	0	0	12
Bubanza	Mpanda	Mpanda	Pilot	Musenyi	43.5	14.8	2.1	5.4	0	0	12	46
Bujumbura mairie	Zone nord	Buterere	National	Buterere II	NA	22.1	10.7	NA	8.1	NA	54	78
Bujumbura mairie	Zone sud	Kanyosha	National	Ruziba II	NA	1.3	0.3	NA	0.6	0	6	48
Bujumbura rural	Bujumbura rural	Kabezi	National	Magara II	NA	8	25.8	NA	0	2	16	40
Bujumbura rural	Isale	Mutimbuzi	National	Maramvya I	NA	42.1	NA	NA	1	2	38	90
Bururi	Matana	Mugamba	Pilot	Gatwe	0	0	0	0	0	0	0	18
Bururi	Matana	Mugamba	Pilot	Ruko	0	0	0	0	0	0	0	12
Bururi	Matana	Rutovu	Pilot	Condi	0	0	0	0.2	0	0	8	36
Bururi	Matana	Rutovu	Pilot	Gitobo	1	0.3	0.2	0.2	0	0	0	12
Bururi	Rumonge	Buyengero	Pilot	Kizuga/Nkizi	0.3	0	0	0	0	0	0	12
Bururi	Rumonge	Burambi	National	Busaga	NA	3.3	NA	NA	0	NA	2	20
Bururi	Rumonge	Buyengero	Pilot	Mudende	10.7	1.6	0	0	0	0	0	44
Cankuzo	Cankuzo	Cankuzo	National	Muhweza	NA	0	0	NA	0	0	2	44
Cibitoke	Cibitoke	Murwi	Pilot	Mirombero	2	1.6	15.4	0.5	0	4	4	34
Cibitoke	Cibitoke	Rugombo	Pilot	Munyika	30.4	6.3	5	4.1	6.4	20	38	70
Gitega	Gitega	Gitega	National	Rutoke I	NA	0	0.3	NA	0	0	0	16
Karusi	Nyabikere	Gihogazi	National	Ramba	NA	1.7	NA	NA	0.9	0	4	16
Kayanza	Musema	Rango	National	Karehe	NA	0	NA	NA	0	0	0	14
Kirundo	Kirundo	Kirundo	National	Yaranda	NA	1.7	0.7	NA	0	0	24	58
Makamba	Makamba	Kayogoro	National	Mugeni	NA	5.3	19.1	NA	0.9	2	30	64
Makamba	Nyanza-Lac	Nyanza-Lac	National	Kabo	NA	19	13	NA	0.9	16	10	66
Makamba	Nyanza-Lac	Vugizo	National	Vugizo	NA	0.7	NA	NA	1.2	NA	12	42
Muyinga	Muyinga	Muyinga	National	Musenga	NA	0	0.3	NA	0	0	6	36
Mwaro	Kibumbu	Kayokwe	National	Kibogoye II	NA	0.3	0	NA	0	NA	2	32
Ngozi	Ngozi	Busiga	National	Mparamirundi	NA	0	0	NA	0	NA	12	48
Rumonge	Bururi	Rumonge	Pilot	Nyamibu	55.5	20.7	21.6	12.8	9.1	4	26	72
Rumonge	Bururi	Rumonge	Pilot	Rukinga	7.4	0.9	2.3	4.1	3.6	6	30	46
Rutana	Gihofi	Giharo	National	Muzye	NA	3.7	4.5	NA	0	0	0	44
Rutana	Rutana	Musongati	National	Gisuriro	NA	1.3	0.8	NA	0	0	12	66
Ruyigi	Kinyinya	Kinyinya	National	Nyamusasa	NA	3.3	NA	NA	0	0	12.8	46.2
**Total average**					**12.7**	**5.2**	**5.4**	**2.3**	**1.1**	**2.2**	**11.6**	**41.4**

CCA = circulating cathodic antigen point-of-care urine cassette assay; NA = not applicable, the epidemiological survey was not done in this specific year. The pilot study started in 12 schools in 2007 and 19 schools in 2008 (baseline). Children at sentinel schools were assessed annually for *S. mansoni* infection and soil-transmitted helminth infections (Kato-Katz), and the school-level prevalence was obtained.
